# Artificial Intelligence‐Generated Synthetic Data in Healthcare: A False Promise for Underserved Populations?

**DOI:** 10.1111/bioe.70121

**Published:** 2026-05-19

**Authors:** Stéphanie Baggio

**Affiliations:** ^1^ Institute of Psychology University of Lausanne Lausanne Vaud Switzerland; ^2^ Institute of Primary Health Care (BIHAM) University of Bern Bern Switzerland

**Keywords:** algorithmic bias, artificial intelligence, informed consent, medical ethics, synthetic data, underserved populations

## Abstract

Artificial intelligence (AI)‐generated synthetic data has emerged as a promising solution to address the underrepresentation of underserved populations in medical AI systems. By artificially generating data that mimics real‐world patient information, proponents argue that AI‐generated synthetic data can fill data gaps, improve algorithmic fairness, and mitigate bias without requiring costly data collection or raising privacy concerns. However, in this article, I challenge this optimistic view. I argue that AI‐generated synthetic data may amplify existing biases rather than mitigate them, and complicate informed consent processes in ways that disproportionately harm underserved populations. I examine two critical ethical dimensions: (1) how the opacity inherent in synthetic data generation can reproduce and amplify discriminatory patterns present in the source data, and (2) how AI‐generated synthetic data complexifies informed consent. I conclude that while AI‐generated synthetic data offers apparent technical advantages, it fails to address, and may worsen, data disparities affecting underserved populations. Rather than pursuing synthetic solutions, I believe that we should address the structural barriers that prevent genuine inclusion of underserved populations in medical research.

## 1 Introduction

Artificial intelligence (AI) has rapidly transformed healthcare, offering powerful tools for disease detection, treatment optimization, and clinical decision support [1, 2]. However, medical AI systems also raise significant ethical challenges. A major concern is that these systems may perpetuate and amplify existing health disparities, particularly for underserved populations, that is, groups that face barriers to accessing adequate healthcare and are often excluded from clinical research due to economic, geographic, social, or cultural factors [3].

Central to these challenges is the problem of *opacity*. Medical AI systems, especially when they rely on deep learning algorithms, operate as “black boxes” where neither developers, clinicians, nor patients can fully understand how conclusions are reached [4, 5]. This opacity creates two major ethical problems. First, it could enable the hidden amplification of bias against underserved populations, who are frequently underrepresented in training data or subjected to discriminatory patterns reflected in historical medical records [6, 7]. Second, AI opacity undermines informed consent by creating a “double ignorance problem” in which neither patients nor clinicians understand how AI systems work, what their limitations are, or how they might fail for specific populations [8].

AI‐generated synthetic data has emerged as an apparently promising solution. Synthetic data consists of artificially generated information created by machine learning algorithms that mimics real patient data without involving actual individuals [9]. Proponents of synthetic data argue that it can address multiple problems simultaneously: filling gaps where underserved populations are underrepresented, reducing privacy concerns, eliminating the need for difficult data collection, and potentially mitigating algorithmic bias [10].

Although some scholars have expressed concerns about AI‐generated synthetic data [9, 10], such technical solutions are gaining traction in the development of medical AI. This warrants critical ethical scrutiny. In this article, I expand upon existing ethical considerations and contend that, rather than resolving the ethical issues arising from AI opacity, AI‐generated synthetic data may exacerbate them, particularly for underserved populations. More specifically, I will examine how the technical characteristics of AI‐generated synthetic data create distinct ethical problems that compound existing inequities and affect informed consent. I will examine two critical ethical dimensions. First, I will demonstrate how synthetic data generation can amplify rather than mitigate existing biases through the same opaque mechanisms that create problems in medical AI systems. Second, I will analyse how synthetic data complicates informed consent by adding new layers of opacity and raising concerns about autonomy and representation. I will conclude that synthetic data is insufficient to address unjust data disparities and may create an illusion of objectivity that diverts attention from necessary structural reforms. Before delving into the ethical problems, the next section introduces the specificities and methods of AI‐generated synthetic data.

## 2 Understanding AI‐Generated Synthetic Data

The use of synthetic data in healthcare is not new. Researchers have long employed synthetic data in simulation studies to test hypotheses by manipulating parameters, such as modelling virus transmission rates or disease progression. Traditional synthetic data has also been used on a limited scale to supplement real‐world data sets, particularly for imputing missing values. When researchers know certain characteristics of an individual but lack specific data points, they can estimate missing information using data from comparable individuals.

However, contemporary AI‐generated synthetic data represents a fundamental shift. This includes synthetic data produced using modern deep learning methods, such as generative adversarial networks (GANs) and variational autoencoders (VAEs). These methods are becoming increasingly important in medical AI applications. Traditional synthetic data was created using human‐designed rules and replicable statistical models, applied on a limited scale to meet specific analytical needs. This is for example the case of classification and regression trees (CART), random forests, and Bayesian networks. Although these methods can produce complex synthetic datasets, they operate using explicit decision rules and conditional probability structures that can be traced and audited. For instance, a decision tree generates synthetic data through a series of interpretable branching decisions based on feature thresholds. Similarly, a Bayesian network encodes relationships through explicit conditional dependencies that can be inspected and validated. In contrast, AI‐generated synthetic data uses opaque machine learning algorithms that learn complex patterns from real data and generate entirely new synthetic participants on a massive scale [10]. These algorithms employ the same deep learning techniques that create opacity in medical AI systems, which I mentioned in the introduction, that is, techniques where even developers cannot fully explain how the system reaches its outputs. For example, a researcher studying disease prevalence might discover that participants from socioeconomically deprived areas constitute only 0.5% of their dataset, despite representing 15% of the target population. Using GAN, the researcher can generate synthetic participants that supposedly mirror the characteristics of deprived populations. The algorithm infers not only demographic information (housing density, income) but also complex behavioural patterns, social interactions, health trajectories, and disease characteristics. As with deep learning systems already in use in medical AI systems, understanding exactly how the model creates these synthetic participants is impossible.

The increasing adoption of AI‐generated synthetic data stems from several apparent advantages [9, 10], as summarised in Table 1.

**Table 1:** Apparent advantages of AI‐generated synthetic data.

Topic	Description
Reduced human error	Avoids human errors, incomplete data, or misunderstandings
Cost and speed	Eliminates expensive and time‐consuming data collection
Data scarcity	Enables research when little real‐world data exists
Diversity	Can theoretically generate data representing diverse populations
Informed consent	Appears to eliminate consent requirements for data without real individuals
Privacy protection	Involves no actual individuals, reducing privacy risks
Security	No risk of breaches involving actual patient information

These potential advantages make synthetic data particularly attractive for addressing underrepresentation of underserved populations in medical research. Rather than confronting the structural barriers that exclude these populations from research participation, synthetic data offers an apparently simple technological fix. However, this appeal obscures serious ethical problems. The first problem, presented in the next section, is that it may amplify rather than mitigate bias.

## 3 Amplifying Rather Than Mitigating Bias

The first major ethical limitation of AI‐generated synthetic data is that it can reproduce and amplify the very biases it is designed to address [9]. Although bias amplification in medical AI systems is well documented [3, 6, 7], the specific mechanisms by which AI‐generated synthetic data reproduces and amplifies these biases for underserved populations require closer examination. Existing literature has raised concerns about the limitations of synthetic data [9, 10], but it has not yet fully analysed the ethical issues of AI‐generated synthetic data. This problem arises from the way synthetic data is generated, which mirrors the hidden bias amplification that occurs in medical AI systems more broadly. Medical AI algorithms learn from source data using recursive learning strategies rather than simply reflecting data passively. These strategies can strengthen certain associations between patient characteristics and outcomes or assign greater weight to features present in the source data. When source data contains biases, such as underrepresentation of underserved populations or reflection of discriminatory practices, the algorithm interprets these patterns as valid medical knowledge rather than recognizing them as biases [3, 7].

For underserved populations, two primary sources of bias are particularly problematic. First, these populations are frequently underrepresented in training data due to barriers to healthcare access and research participation. If a medical AI system has not been developed using data from low‐income patients, for example, it cannot perform accurately or safely for them. Indeed, they may present with different disease patterns, more untreated comorbidities, or more severe illness at hospital admission. Second, source data often reflects historical discrimination and unequal treatment. When Black patients are systematically denied expensive care or admitted to intensive care later than white patients, AI systems may learn higher thresholds for intervention, thereby perpetuating and potentially amplifying existing disparities [6].

One critical problem is that AI opacity makes this amplification undetectable. Because the algorithms operate as black boxes with constantly learning and adapting models, it is “extremely challenging to understand and estimate how the bias is being amplified” [7, p. 709]. The amplification itself emerges from recursive learning processes but becomes hidden because of opacity.

AI‐generated synthetic data could inherit and amplify these problems. Indeed, its generation employs the same opaque machine learning techniques that create bias amplification in medical AI systems. When synthetic data is created to represent underserved populations, the algorithm learns from existing data that already contains biases through underrepresentation, discrimination, or systematic exclusion. The synthetic data generation process will learn these biased patterns and potentially amplify them through the same recursive learning mechanisms.

As Susser et al. [10, p. 9] observe, “By definition, synthetic data is only an approximation of the measurement data it imitates.” The machine learning algorithms used are neither neutral nor fully controllable [9]. Therefore, although AI‐generated synthetic data is intended to reduce bias, it could amplify it. Due to algorithmic opacity, identifying potential amplified bias is extremely challenging.

A second critical concern is that synthetic data may lack realism or create unrealistic diversity when attempting to represent populations absent or poorly represented in source data. Research in other domains demonstrates that accurate predictions cannot be made outside the scope of source datasets. For example, Sun et al. [11] showed in weather forecasting that extrapolation from well‐documented weak tropical cyclones to poorly‐documented strong cyclones is not possible: The algorithm simply cannot accurately model phenomena absent from its training data. To my knowledge, this limitation has not been adequately studied in healthcare applications of synthetic data. However, I believe that AI‐generated synthetic data likely cannot realistically depict health patterns or personal characteristics that are absent or poorly represented in original source data. When underserved populations are underrepresented in source data, synthetic generation cannot create accurate representations. It can only create algorithmically generated approximations based on biased patterns from other populations. This creates a particularly insidious form of bias because AI‐generated synthetic data can appear more equitable than the original dataset while actually misrepresenting underserved populations [12]. Doctors and researchers may believe they are using fair, representative data when in fact the synthetic data amplifies existing disparities in unknown ways. This could create an “illusion of objectivity” in AI systems. Indeed, technical complexity creates an appearance of scientific validity and neutrality, even when outputs are biased [12]. Healthcare providers and researchers tend to place greater trust in technological decisions compared to human ones [7], believing that bias has been addressed and that outputs represent objective, value‐free results. For synthetic data, researchers and clinicians may believe that using synthetic data has solved the problem of underrepresentation, discouraging the difficult but necessary work of addressing structural barriers to genuine inclusion in data collection.

One might object that synthetic data has long been used in healthcare for imputing missing values, and this practice also risks bias and misrepresentation of underserved populations, who disproportionately have missing data [13]. However, I think that there are significant differences between traditional imputation and AI‐generated synthetic data. First, traditional imputation methods have been extensively studied prior to implementation. A PubMed search conducted in June 2025 yielded over 1500 publications dating from 1989, including multiple systematic reviews evaluating accuracy and reliability. Second, imputation is routinely complemented by sensitivity analyses that test whether conclusions remain consistent when different methodological approaches are used. If findings are inconsistent across imputation methods, researchers can identify weaknesses and adjust their conclusions accordingly. In contrast, AI‐generated synthetic data lacks both an established validation framework and meaningful sensitivity analyses. Unlike imputation, which fills gaps in existing real‐world datasets using statistically grounded methods, AI‐generated synthetic data creates entirely synthetic participants whose characteristics may not accurately reflect true population distributions. This makes bias detection more difficult and sensitivity analyses less meaningful.

A second objection is that opacity prevents us from identifying whether the bias is amplified. Indeed, opacity cuts both ways: if we cannot trace how bias is amplified, then we cannot confirm whether or not it is amplified in any given case. In theory, opaque synthetic data generation could maintain or even reduce bias in certain situations. However, I would argue that this epistemic uncertainty itself also constitutes an ethical problem. Even if we cannot be sure that bias is amplified, we cannot guarantee that it is not. As bias amplification can have harmful consequences, such as misdiagnosis, inappropriate treatment, and systematic exclusion from effective care, I believe that this uncertainty is not neutral.

Overall, I believe that research into the extent of bias in AI‐generated synthetic data and methods for addressing it is needed before these techniques can be implemented in healthcare. While synthetic data appears promising, we should not rush to adopt it without adequate safeguards, particularly given the potential harm to underserved populations. The next section examines the second major ethical problem: How AI‐generated synthetic data complicates informed consent.

## 4 Complicating Informed Consent

Informed consent is a cornerstone of medical ethics, rooted in the fundamental bioethical principle of autonomy [14]. The challenges surrounding informed consent in the context of medical AI have been well documented [15, 16]. However, the specific ways in which AI‐generated synthetic data exacerbates these challenges, particularly for underserved populations, require further attention. For instance, the impact of the layered opacity of synthetic data generation on the creation of distinct consent pathways and the imposition of disproportionate burdens on marginalised populations has not yet been thoroughly investigated. Individuals have the right to self‐determination and to decide which medical procedures they undergo, and which research they participate in. For consent to be valid, patients must have decision‐making capacity, be able to make voluntary decisions, receive sufficient information, and understand what they are consenting to [15, 16]. The World Health Organization [17, p. 26] explicitly addresses informed consent for AI in healthcare:

AI technologies should not be used for experimentation or manipulation of humans in a health‐care system without valid informed consent. The use of machine‐learning algorithms in diagnosis, prognosis and treatment plans should be incorporated into the process for informed and valid consent.

This means informed consent is required both when medical AI systems are used in clinical care and when they are employed in medical research. However, the opacity of medical AI systems creates what I call a “double ignorance problem”, with neither patients nor clinicians being able to fully understand how these systems work, how they reach conclusions, or what their limitations are [8]. This opacity undermines the understanding necessary for valid informed consent.

At first glance, AI‐generated synthetic data appears to solve informed consent problems because it does not involve real individuals. However, this analysis is superficial. Informed consent is required at two distinct stages when synthetic data is used. First, individuals whose real data is used to train synthetic data generation algorithms should consent to this use. This is not a trivial consent because their data is being used to create algorithmic representations of entire populations. Patients must understand not just that their data will be used for research, but that it will be used to algorithmically generate representations of others, potentially including members of underserved populations to whom they may bear little resemblance. Second, when medical AI systems trained on synthetic data are used to inform diagnosis, prognosis, or treatment decisions, patients should consent to receiving care influenced by these tools. They need to understand that recommendations are based partly or entirely on artificially generated data rather than real patients.

I argue that using AI‐generated synthetic data increases the layers of opacity, thereby exacerbating the double ignorance problem. Clinicians and patients now face not just the opacity of medical AI systems' decision‐making processes, but also the opacity of how synthetic data is generated. Patients whose data is used to create synthetic data are supposed to consent without fully understanding the synthetic generation process. Patients receiving treatment plans based on synthetic data cannot understand what medical recommendations are truly based on.

As with medical AI systems generally, doctors cannot adequately explain how synthetic data is created, which real patient characteristics inform which synthetic patient features, or how the algorithm decides what constitutes realistic representations of underserved populations. This opacity further complicates estimation of risks and limitations, which were already poorly understood when medical AI systems used only real data [8]. Consequently, I believe that the principle of autonomy is violated, as patients cannot meaningfully understand what they are consenting to [15].

This problem exists for all patients but is particularly acute for underserved populations. Indeed, underserved populations already face significant barriers to informed consent compared to the general population. These barriers include language and cultural differences that hinder effective communication, educational disparities and health illiteracy that restrict understanding of medical information, and historical marginalization that fosters mistrust of healthcare providers [16, 18, 19]. A well‐educated patient may question an AI diagnosis, request a second opinion, or independently research the technology. In contrast, a patient from an underserved population may feel less empowered to challenge medical authority, feel ashamed of technical illiteracy, or lack the educational background or language proficiency to request explanations. Moreover, underserved populations may experience the burden of poverty, which limits their capacity to divert cognitive resources away from coping with stressful life circumstances [20]. For these populations, AI opacity combined with synthetic data generation adds another layer of complexity to an already challenging healthcare landscape.

I think this could even create a form of “intervention‐generated inequality”, where healthcare interventions disproportionately benefit less disadvantaged groups [21]. Potential consequences include increased mistrust of healthcare, delayed care‐seeking behaviour, reduced treatment adherence, and disengagement from healthcare systems. These outcomes directly contribute to health disparities by creating additional barriers to healthcare access.

A fundamental ethical concern is that AI‐generated synthetic data for underserved populations is created without input from those populations [22]. Medicine has evolved from paternalistic models where doctors made decisions for patients without consultation, toward models of shared decision‐making that respect patient autonomy and agency. However, the use of medical AI systems, particularly with undisclosed synthetic data, threatens to reverse this progress, creating what Savulescu et al. [8, p. 153] term “machine paternalism.” Artificially generating data to represent underserved populations bypasses their agency and preferences entirely. This is particularly problematic given that these populations have historically experienced paternalism and struggled to assert autonomy within healthcare settings.

One might object that underserved populations are difficult to reach, and synthetic data at least attempts to improve their representation in research from which they are often excluded. However, it is unclear whether synthetic data accurately represents these populations. Creating a false impression of inclusion through synthetic data may be worse than acknowledging gaps, as it diverts resources and attention from addressing the structural barriers that prevent genuine participation.

A potential objection to my critique of AI‐generated synthetic data is that traditional analytical practices, such as imputing missing data, raise similar informed consent concerns without generating equivalent ethical scrutiny. As Randall et al. [22, p. 5] observe: “If someone declines to report their race or ethnicity in a survey, later generating that value through advanced analytical methods overrides that initial refusal.” However, this objection overlooks critical differences in scope and scale. Imputation addresses small gaps in existing real‐world data by estimating missing information for actual participants who consented to research participation. In contrast, AI‐generated synthetic data creates entirely new artificial patients on a massive scale, that is, patients who never existed, never consented, and whose characteristics are algorithmically inferred from other populations. Moreover, even if traditional practices raise autonomy concerns, this does not justify adding new practices with similar or worse problems. Rather, the ethical issues with synthetic data should prompt us to reconsider our analytical practices with respect to patient autonomy and data use.

## 5 Conclusion

AI‐generated synthetic data has emerged as an apparently promising solution to the underrepresentation of underserved populations in medical research. By artificially generating data that mimics real patient information, proponents argue that synthetic data can improve algorithmic fairness and mitigate bias without the challenges of genuine data collection. However, my ethical analysis shows that this promise could be false, or at least, overstated. Synthetic data may amplify rather than mitigate existing biases through the same opaque machine learning mechanisms that create problems in medical AI systems. When source data contains biases through underrepresentation or discrimination, synthetic data generation can learn and potentially amplify these patterns, making them difficult to detect due to algorithmic opacity. This can result in hidden misrepresentation of underserved populations that appears equitable but perpetuates disparities.

Furthermore, synthetic data complicates rather than eliminates informed consent requirements. Patients whose data trains generation algorithms and patients receiving AI‐informed care both require valid consent, yet the double opacity inherent in synthetic data (opacity in both data generation and clinical decision‐making) undermines the understanding necessary for autonomous decision‐making. This disproportionately harms underserved populations who already face barriers to informed consent due to educational disparities, language barriers, and historical marginalization.

Synthetic data offers the superficial appearance of addressing these problems without confronting their root causes. Worse, by creating the illusion that underserved populations are now “represented” in data sets, synthetic data may reduce pressure to address structural barriers to genuine inclusion. As Shanley et al. [9, p. 2154] correctly observe:

Synthetic data is often presented as a promising tool to reduce knowledge or training gaps related to underrepresented groups thereby achieving diversification. However, synthetic data is not a magic fix to ensure equitable results.

As AI becomes increasingly prevalent in healthcare, we must ensure that its implementation advances rather than undermines equity. This requires recognizing that some problems cannot be solved through technological innovation alone. They require sustained commitment to social justice, structural change, and meaningful inclusion of underrepresented communities.

## Conflicts of Interest

The author declares no conflicts of interest.

## Data Availability

The author has nothing to report.
